# Effects of Edible Treats Containing *Ascophyllum nodosum* on the Oral Health of Dogs: A Double-Blind, Randomized, Placebo-Controlled Single-Center Study

**DOI:** 10.3389/fvets.2018.00168

**Published:** 2018-07-27

**Authors:** Jerzy Gawor, Michał Jank, Katarzyna Jodkowska, Emilia Klim, Ulla K. Svensson

**Affiliations:** ^1^Klinika Arka Krakow, Krakow, Poland; ^2^Division of Pharmacology and Toxicology, Department of Preclinical Sciences, Faculty of Veterinary Medicine, Warsaw University of Life Sciences, Warsaw, Poland; ^3^Department of Small Animal Diseases, Faculty of Veterinary Medicine, Warsaw University of Life Sciences, Warsaw, Poland; ^4^Klinika Puławska Warszawa, Warsaw, Poland; ^5^UKS Life Science Consulting AB, Lund, Sweden

**Keywords:** plaque index, dental calculus, *Ascophyllum nodosum*, homecare, oral health, oral hygiene, volatile sulfur compound, dogs

## Abstract

The objective of this placebo-controlled, double-blind, randomized study (designed according to evidence based medicine standards) was to determine the effect of 90-day administration of edible treats containing the brown algae, *Ascophyllum nodosum*, on plaque and dental calculus accumulation on the teeth of dogs, as well as on other parameters characterizing canine oral health status, including: plaque index (PI), calculus index (CI), oral health index (OHI), gingival bleeding index (GBI), and volatile sulfur compound (VSC) concentration. Sixty client-owned dogs, including Japanese chin, miniature Schnauzer, Chihuahua, Pomeranian, and West Highland White Terrier (WHWT) breeds, underwent professional dental cleaning and were randomly subdivided into two groups receiving daily edible treats containing the brown algae *A. nodosum*, or placebo, adjusted to their bodyweight. After a comprehensive oral health assessment, including a professional dental cleaning, which were both performed under general anesthesia, clinical assessments of PI, CI, OHI, GBI, and VSC concentration were performed under sedation after 30, 60, and 90 days of treatment. Oral administration of edible treats containing *A. nodosum* significantly improved PI, CI, and VSC concentration, compared with the placebo-treated group. The consumption of edible treats containing *A. nodosum* efficiently decreased plaque and calculus accumulation in the investigated dogs. Dogs treated with *A. nodosum* also exhibited significantly lower concentrations of VSC and better oral health status (e.g., OHI and GBI) than those in the placebo-control group.

## Introduction

Periodontal diseases, which are the most common oral diseases in both humans and dogs (1), result in inflammatory destruction of periodontal ligaments and alveolar bone, with or without gingival recession and/or pocket formation (2). Numerous factors contribute to the prevalence and severity of periodontal disease in dogs (3). A higher susceptibility to naturally occurring periodontal disease has been observed in small-breed dogs and many animals affected by periodontal disease exhibit alterations in their vital organs (4–8).

Periodontal disease pathogenesis initiates via the formation of bacterial plaque which, following mineralization, becomes calculus. Subsequently, the unique inflammatory responses of host immune systems to the plaque determine the progress of periodontal disease (9).

The rough surface of calculus becomes a substrate for further plaque accumulation, and dental deposits enlarge significantly over time. This process, in which dental plaque plays a critical role, is the main cause of periodontal disease in dogs (10). This condition also results in significant changes in the oral microbiome composition. The results of recent studies show that healthy canine plaque is dominated by gram-negative bacterial species, whereas gram-positive anaerobic species predominate during disease (11–14).

Strategies for the prevention of periodontal disease are based on the control of plaque and calculus formation. Such control is established by the combined efforts of veterinarians and pet owners, who must consider what is feasible for specific pets and achievable by the owner. Oral cavity home care can generally be divided into active and passive methods. Among active modalities, the most effective method is daily tooth brushing, which can result in plaque reduction of 37.4% and an 80.2% reduction of calculus compared with that in dogs with non-brushed teeth (15). Other active methods include application of oral cleansing gels or barrier sealants.

The compliance of clients in performing the most effective methods of oral hygiene varies from 53% (16), reported 6 months after periodontal treatment, to an overall rate of 1% (17). Low compliance is caused by factors such as manual difficulties, a non-collaborative attitude of patients, or lack of time. For this reason, alongside active home care methods, numerous passive methods, including diet, treats, chews, water additives, and nutritional supplements, have emerged. The effectiveness of passive methods relies on their mechanical or chemical features, or a combination of both (18).

One such product is an edible treat supplemented with the brown alga, *Ascophyllum nodosum*, (ProDen PlaqueOff® Dental Bites, Sweden). Controlled studies performed by two different research groups demonstrated a positive effect of this product in plaque and calculus reduction in dogs and cats, as well as improvements in oral health (19, 20); however, these studies were not double-blind nor randomized. In humans, the effectiveness of an *A. nodosum*-based supplement was evaluated in a randomized controlled study; the results indicated significantly reduced plaque, calculus, and gingivitis indices (21).

The objective of the present study was to determine the influence of 90-day administration of edible treats containing *A. nodosum* [ProDen PlaqueOff®, Sweden] on plaque and dental calculus accumulation, in addition to other parameters characterizing oral health status, including oral health index (OHI), plaque index (PI), calculus index (CI), gingival bleeding index (GBI), and volatile sulfur compound (VSC) concentration, in canine oral cavities. The study design was double-blind, randomized, placebo-controlled, and single-center.

## Materials and methods

### Animals

Animals included in the study were client-owned and all procedures performed during the study were conducted according to standard veterinary care procedures and following Polish Regulations (Art.1 ust.2 pkt1 Dz. U.2015 poz. 266). Ethics committee approval was not required. All dog owners signed approval for the participation of their dogs in the study.

### Inclusion/exclusion criteria

Dogs were excluded from the study if they had received treatment with NSAIDs, other anti-inflammatory drugs, or antibiotics within the previous 30 days. Included dogs were required to have the majority of their teeth, including (bilaterally) maxillary I3, C, P3, P4, and M1, and mandibular C, P3, P4, and M1. The oral health status of dogs was required to be no worse than PD2 (mild), according to American Veterinary Dental College (AVDC) staging (www.avdc.org). Dental health status of all dogs was evaluated by the same scorer (JG) (22). To ascertain the general health status of dogs that qualified for the study, the following parameters were analyzed: urine, complete blood count (CBC), thyroxine, serum tests (UREA, CREA, TP, ALB, ALT, ALKP, and GLU), and clinical assessment (see section Dental Procedures and Oral Health Assessment, “*Dental procedures and oral health assessment*”). Any dogs with systemic disease, or that were pregnant, or had increased thyroxine levels were excluded.

Randomization lists for each trial and each group were generated using the randomization program, Research Randomizer (23). The numbers of dogs receiving active substance or placebo was set to equal, with each pair of participants added to the study. Clients, personnel conducting the clinical study, and the statistician were blinded to products administered. Finally, the population of dogs was randomly divided into two equal groups, each containing 30 animals.

None of the dogs received any oral hygiene treatments during the study, other than as described in this manuscript.

### Products

During the study, all dogs received cereal edible treats prepared by the manufacturer of the investigated product (Sweden Care AB). One group of dogs received edible treats containing *A. nodosum* (25% w/w) (P2) whereas the second group received the same bites without any alga (P1). The edible treats weighed 0.2 g and the daily dosage was 1 dental bite/kg dog body weight, where each bite contained 0.05 g of algae. The two products (active and placebo) were packaged in neutral non-transparent bags. Bags were labeled with the study details and the date, but were blinded for the personnel who participated in the clinical trial, and the statisticians.

During the study period, all dogs were fed dry kibble (Hill's SP Canine Adult Chicken) and tap water was provided *ad libitum*. Information about food intake, including any variations, was recorded by the dog owners.

### Dental procedures and oral health assessment

Animals qualified for anesthesia on the first day of the study (T0) if they had an anesthesia risk of ≤1 (24). All dogs were first evaluated by performing a general physical examination, which included visual assessment, heart and lung auscultation, capillary refill time assessment, body weight, and body temperature measurements. All parameters and remarks were recorded on an anesthesia chart. Intravenous catheters were placed and blood collected for immediate laboratory work. Urine samples were collected by cystocentesis and urine immediately evaluated in a UA analyzer to measure pH and specific gravity, and test for protein, glucose, ketones, and blood.

The OHI of each dog was evaluated before sedation, according to published protocols, and was defined as the sum of scores obtained for three parameters: lymphadenopathy, dental deposits, and periodontal disease, with 0 points indicating optimal oral health, and 6 points indicating the poorest oral health (25).

Sedation was performed using recommended doses of medetomidine (Sedator, Eurovet Animal Health B.V.) and butorphanol (Torbugesic, Zoetis, Polska Sp. z o.o.). Then, pre-oxygenation was performed using a mask for the delivery of medical oxygen. After sedation was achieved, animals received propofol (Scanofol, ScanVet, Poland Sp. z o.o.) at recommended doses, to induce general anesthesia. An endotracheal tube was placed, the cuff filled, cardio monitor peripheral probes attached to the animal's body, and a temperature maintenance system installed. General anesthesia was maintained using isoflurane and oxygen for the duration of the dental procedure.

The following procedures were performed under general anesthesia: comprehensive oral health assessment with periodontal probing, dental charting, and full mouth radiography, followed by professional dental cleaning, which included descaling and polishing the teeth. Mechanical descaling of supra- and sub-gingival calculus and plaque using an ultrasonic scaler was followed by delicate flushing of the mouth with water and polishing of the crowns using powdered pumice without fluoride. IC plaque disclosing solution (IC plaque IM3) was used to confirm the complete removal of deposits. Any remaining deposits were removed with repetition of the entire procedure (descaling, flushing, and polishing). At this stage, any dog with stage 2 periodontal disease or greater, or with an oral tumor, contact mucositis, was excluded from the study.

The same procedure of physical, OHI assessment, and sedation, but not general anesthesia, blood work and urinalysis, was performed again 30, 60, and 90 days (T30, T60, and T90, respectively) after T0. After sedation, dogs were first evaluated for VSC levels using OraStrip (OraStrip QuickCheck PDx BioTech). The testing pad of the strip was slid gently along the entire maxillary facial (buccal and labial) gingival margin for 10 s and the color of the pad recorded.

After assessment of gingivitis, plaque and calculus coverage was measured on nine target teeth: maxilla, I3, C, P3, P4, and M1; and mandible: C, P3, P4, and M1. The buccal surface of target teeth on both sides of the mouth was scored by the same experienced scorer (JG). The previously measured intra-examiner reproducibility levels of this scorer regarding probing depth, PI, and calculus index, were 100, 89, and 96% respectively (26).

Initially, the GBI was evaluated using a periodontal probe (UNC 15, IM3) according to previously described criteria (27). The PI was then scored by disclosing the plaque, using plaque disclosing solution (IC plaque IM3), and quantifying the coverage (CO) and thickness of the plaque (THI) using the modified Logan & Boyce index (28). Combined PI values were calculated by multiplication of CO and THI values. Plaque was not brushed from the surface of the teeth because it was scored three times (T30, T60, and T90), and this scoring required that existing plaque at intermediate scoring times was not disturbed. Finally, the calculus index was evaluated using a dental explorer to determine the edge of the tartar with minimal disturbance of the plaque. The coverage of calculus was scored according to published protocols (29). Every dog had an individual form on which assessment results were recorded. To avoid confusion, data from T30, T60, and T90 were recorded using differently colored charts (yellow, green, and blue, respectively). Body condition was also evaluated using a nine-point scale at each time point (T0, T30, T60, and T90).

### Statistical analyses

All numerical variables are presented as the arithmetic mean ± standard deviation, with range in brackets, or as the arithmetic mean with 95% confidence intervals in figures. Unpaired Student's *t*-tests or one-way analysis of variance (ANOVA) were used for unrepeated comparisons of numerical variables. A Pearson's chi-square test was used for unrepeated comparisons of categorical variables. To compare two groups at the four time points, a repeated-measure ANOVA was used as an omnibus test. Sphericity assumption was verified using Mauchly's test and, if violated, the Greenhouse-Geisser correction was applied. If ANOVA tests yielded significant results, a *post-hoc* analysis was performed using the honestly significant difference (HSD) Tukey's test for equal sample sizes. The significance level (α) was set at 0.05. All analyses were performed in Statistica 12.5 (StatSoft Inc.).

All variables were non-normally distributed (Shapiro-Wilk W test *p* < 0.05). Despite this, a repeated-measure ANOVA was applied as there were three factors that acted in favor of a parametric approach: (1) sample sizes were equal (orthogonal study design); (2) the size of each sample was relatively high (*n* = 30); and (3) variances of all variables at all time points were similar (Levene's test for equality of variances *p* > 0.05, homoscedasticity assumption held).

## Results

### Basic characteristics of dogs included in the study

The study was conducted using 60 dogs: 35 females (58%) and 25 males (42%), representing five breeds: miniature Schnauzer (*n* = 17), Japanese chin (*n* = 8), Chihuahua (*n* = 31), Pomeranian (*n* = 3), and West Highland White Terrier (WHWT) (*n* = 1). The results of clinical assessments and laboratory tests at T0 revealed no significant deviations and were within standard ranges typical for healthy animals, including thyroid assessment by measurement of total thyroxine level. The ages of the dogs ranged from 10 to 130 months, with an arithmetic mean (± SD) of 46.2 ± 26.3 months. Age was similar in both sexes (unpaired t-Student test, *p* = 0.733) and across all breeds (one-way ANOVA, *p* = 0.111).

All dogs underwent full mouth oral assessment and dental radiography which revealed stage 1 disease in 43 dogs and stage 2 disease in 17 dogs, evenly distributed between the randomized groups.

Sexes were evenly divided between the two experimental groups (chi-square test, *p* = 0.432); the P1 group consisted of 19 females (63%) and 11 males (37%), with 16 females (53%) and 14 males (47%) in the P2 group. Age was similar in both experimental groups (unpaired t-Student test, *p* = 0.298); the mean age of dogs in the P1 group was 49.8 ± 27.8 (range 10–130) months, whereas in P2 group it was 42.7 ± 24.7 (range 10–91) months.

**Figure 1:**
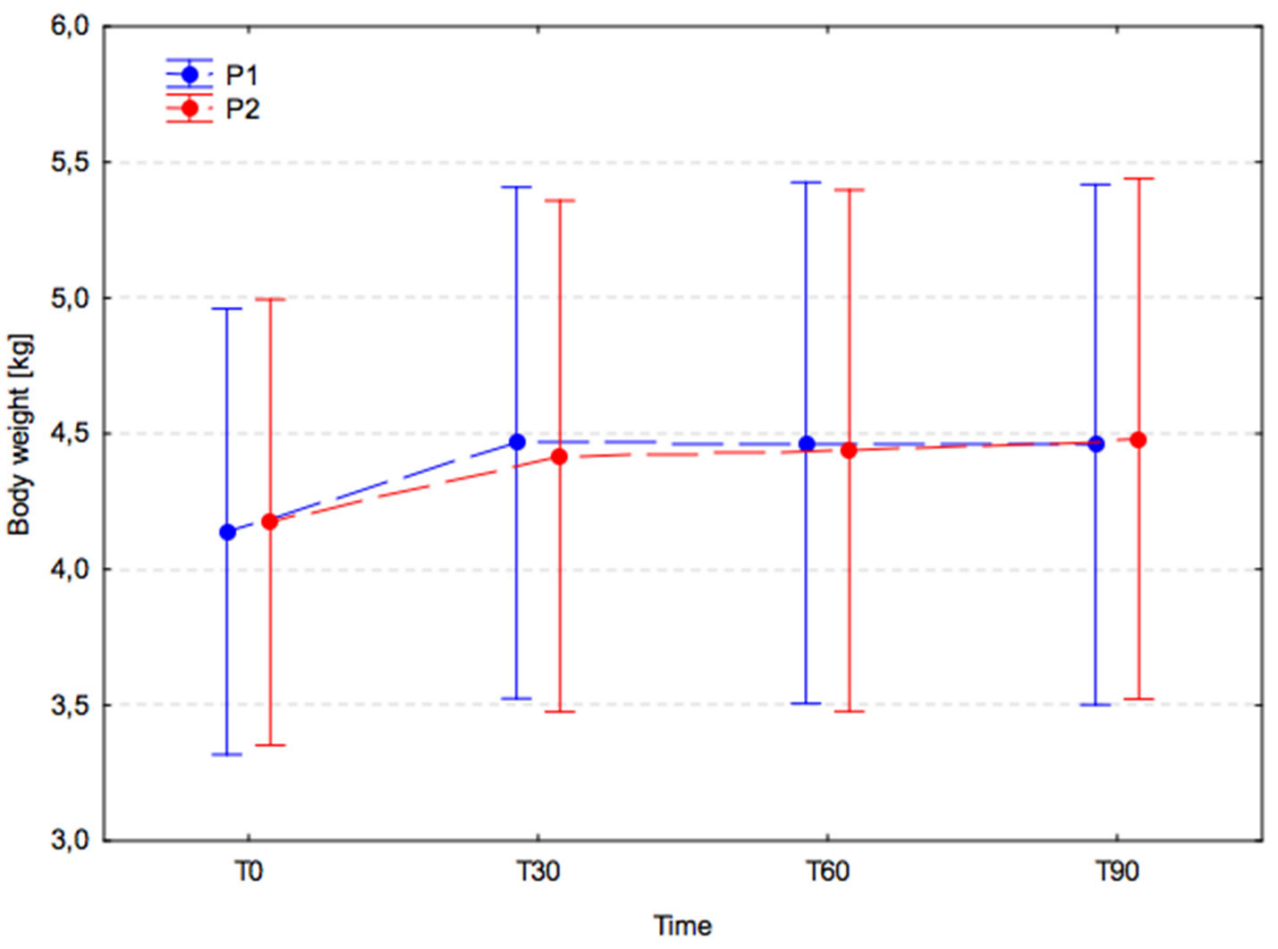
Body weight changes (arithmetic mean with 95% confidence interval) in dogs receiving edible treats, for 90 consecutive days; Placebo group (P1, blue); *A. nodosum* group (P2, red). The dotted lines connect data obtained at the specified time points in each experimental group.

In both groups, a significant increase in body weight was observed between T0 (P1, 4.1 ± 2.3 and P2, 4.2 ± 2.3) and T30 (P1, 4.5 ± 2.7 and P2, 4.4 ± 2.5; P1, *p* < 0.001 and P2, *p* = 0.006), and this remained significantly higher at T60 (P1, *p* < 0.001; P2, *p* = 0.002) and T90 (P1, *p* < 0.001; P2, *p* < 0.001; Figure 1). No significant changes in body weight were observed between T30 and T60 (P1, *p* > 0.999; P2, *p* > 0.999) or between T60 and T90 (P1, *p* > 0.999; P2, *p* = 0.998) in either group. Inter-group comparisons revealed no significant difference between experimental groups (*p* = 0.993) at T0, T30, T60, and T90.

Body condition score increased significantly in both groups between T0 and T30 (*p* = 0.002), and then remained unchanged. During the entire period of the study, the body condition scores of all dogs remained in the range 4–8. Inter-group comparison revealed no significant between-group difference in body condition score at any time point (*p* = 0.656).

### Combined plaque index (PI)

**Figure 2:**
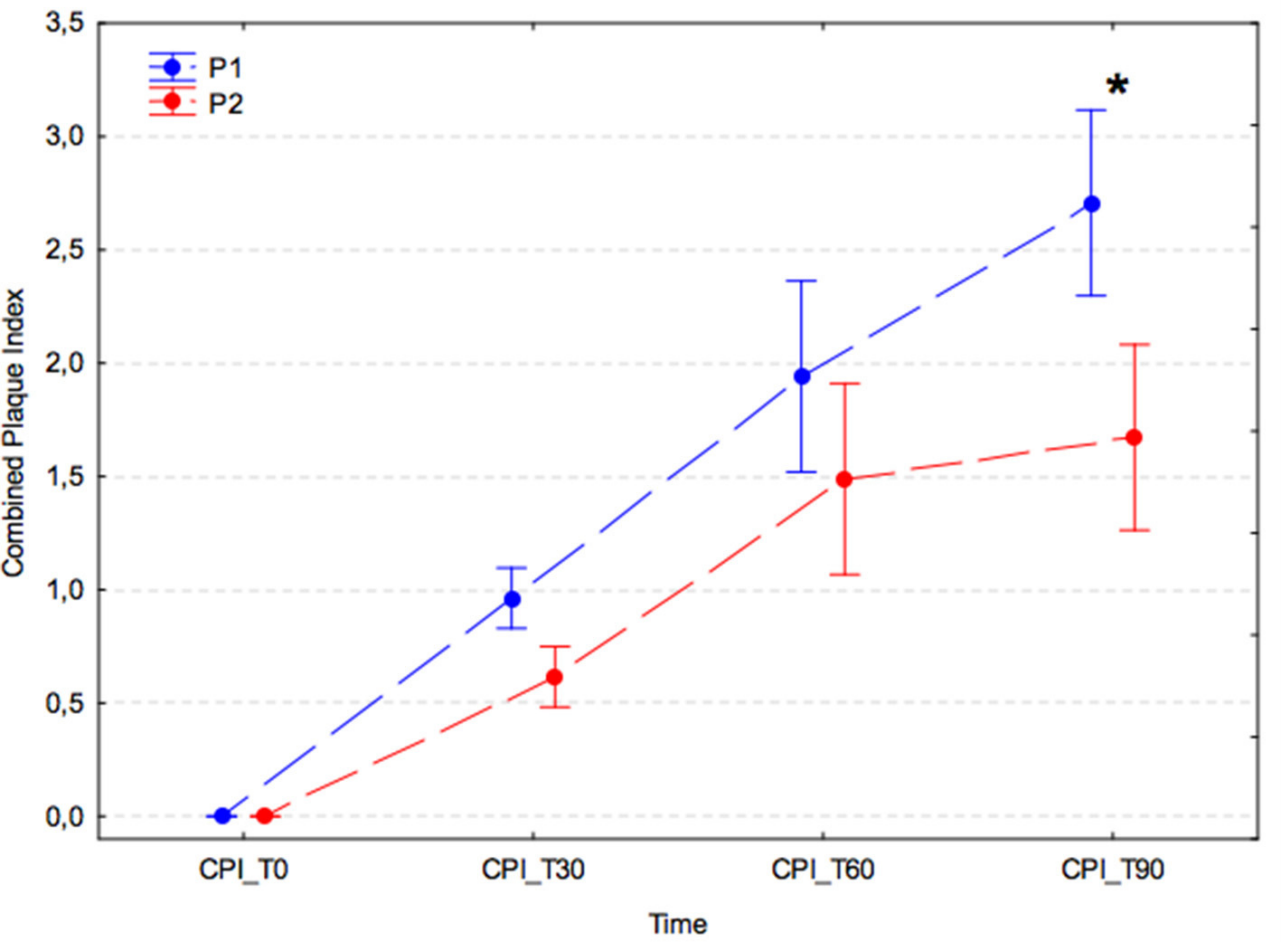
Average combined plaque index (arithmetic mean with 95% confidence interval) for dogs receiving edible treats containing placebo (P1 group, blue) or *A. nodosum* (P2 group, red) for 90 consecutive days. Asterisk (^*^) denotes a significant intergroup difference at a given time point. Dotted lines connect data obtained at the specified time points in each experimental group.

In both experimental groups, there was a significant increase in combined PI at successive time points after the dental procedure (*p* < 0.001); however, in the group receiving *A. nodosum* (P2), the increase in combined PI was slower, and at T90 the combined PI value was significantly lower than that of the placebo group (P1) (P1, 2.71 ± 1.36 vs. P2, 1.67 ± 0.81; *p* < 0.001; Figure 2).

### Calculus index

**Figure 3:**
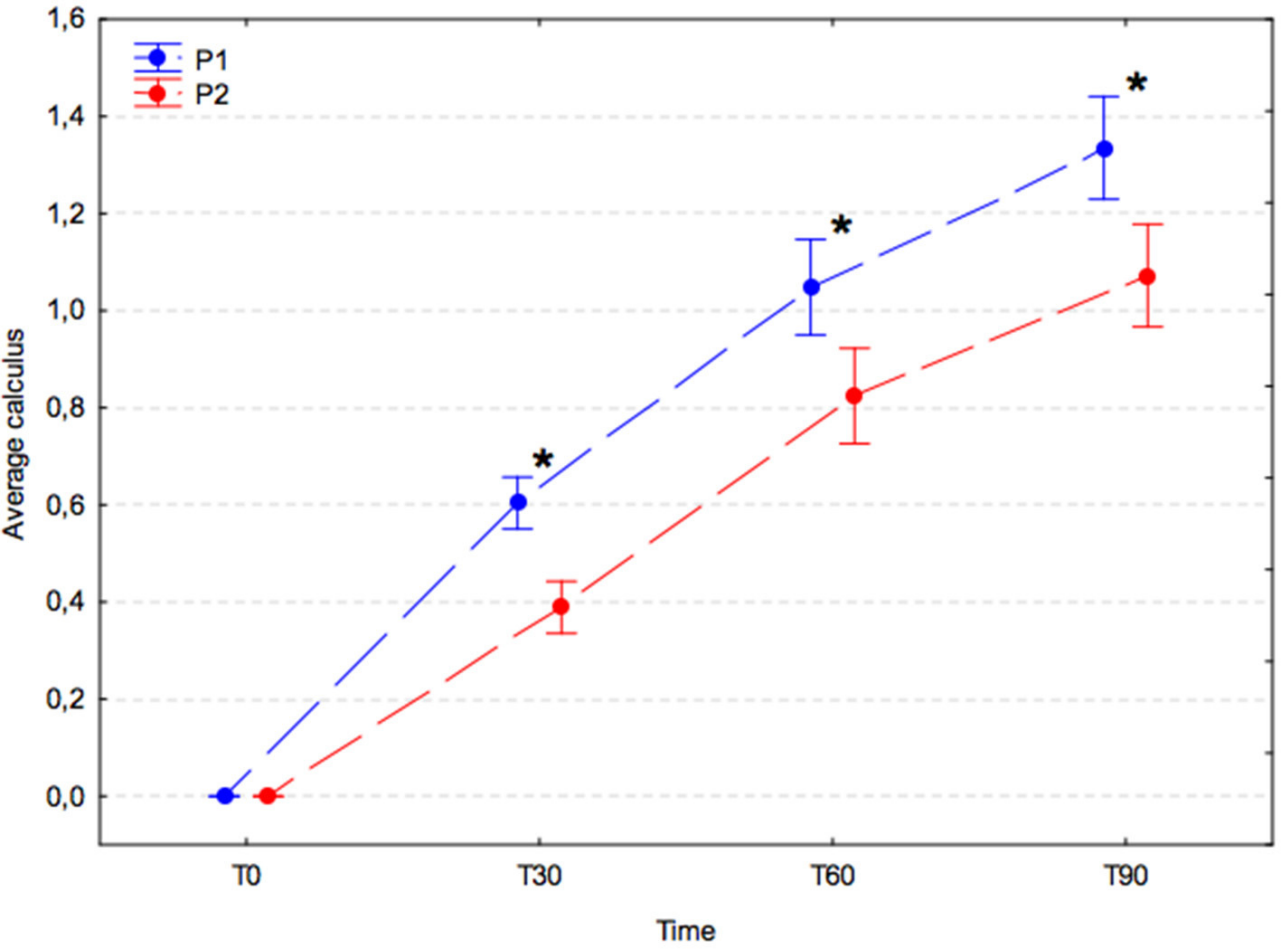
Average calculus index (arithmetic mean with 95% confidence interval) in dogs receiving edible treats containing placebo (P1 group, blue) or *A. nodosum* (P2 group, red) for 90 consecutive days. Asterisk (^*^) denotes a significant intergroup difference at a given time point. Dotted lines connect data obtained at the specified time points in each experimental group.

The average calculus index increased significantly with time in both experimental groups (*p* < 0.001); however, the increase in the placebo group (P1) was significantly greater compared with that in the *A. nodosum*-treated group (P2) at T30 (P1, 0.60 ± 0.15 vs. P2, 0.39 ± 0.14; *p* = 0.002), T60 (P1, 1.05 ± 0.24 vs. P2, 0.82 ± 0.29; *p* = 0.001), and T90 (P1, 1.34 ± 0.23 vs. P2, 1.07 ± 0.39; *p* < 0.001; Figure 3).

### Volatile sulfur compound (VSC) concentration (measured by orastrip)

**Figure 4:**
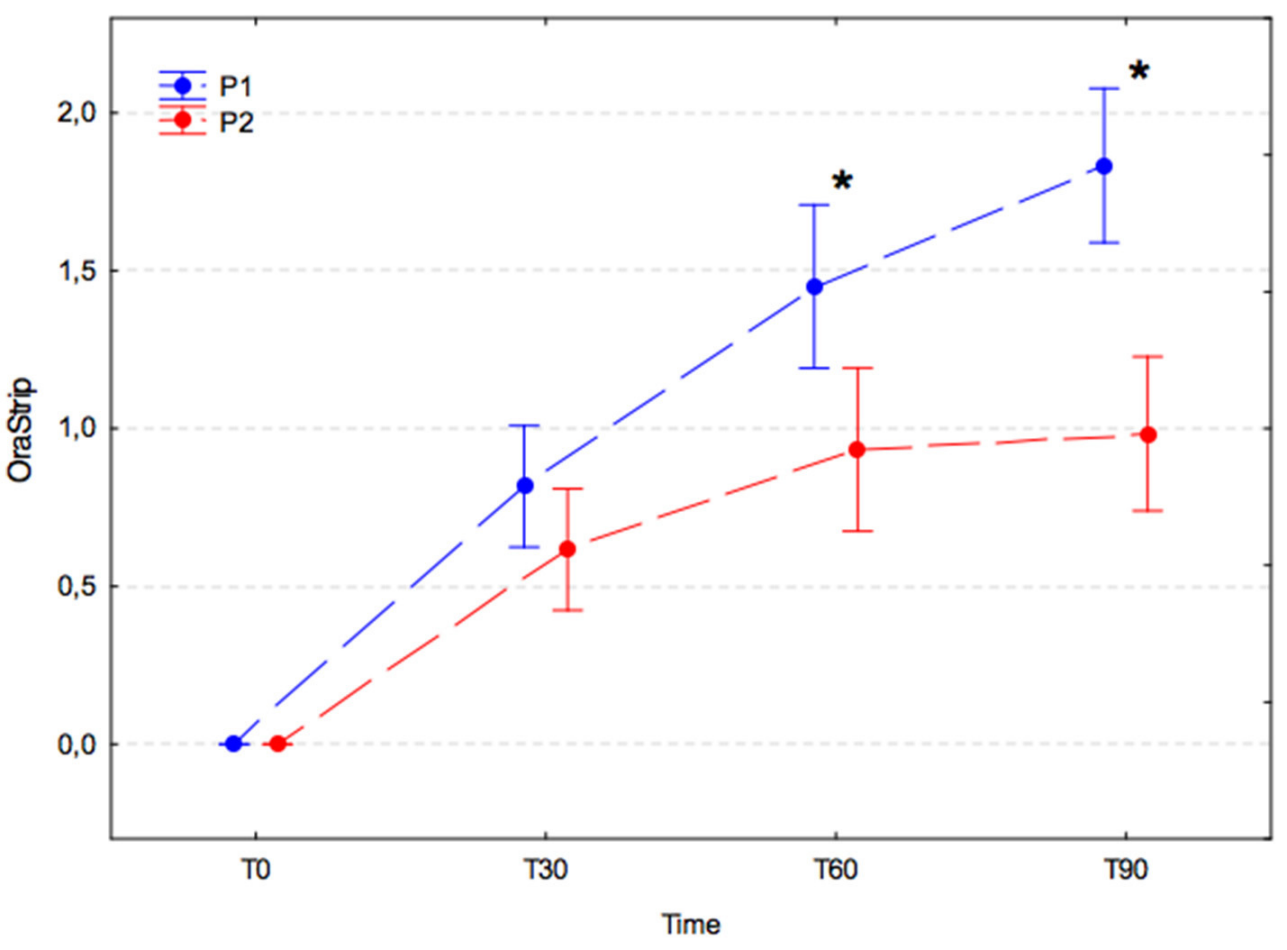
Volatile sulfur compound levels (arithmetic mean with 95% confidence interval) in dogs receiving edible treats containing placebo (P1 group, blue) or *A. nodosum* (P2 group, red) for 90 consecutive days. Asterisk (^*^) denotes significant intergroup difference at a given time point. Dotted lines connect data obtained at the specified time points in each experimental group.

VSC levels increased significantly during the 30 days after the dental procedure in both experimental groups, and values were not significantly different between groups at T30 (*p* = 0.858). Subsequently, the VSC levels in each group changed in different ways. In the P1 group, the VSC level was found to have increased significantly on subsequent measurements at T60 and T90 (*p*-values, T0 vs. T30, *p* < 0.001; T30 vs. T60, *p* < 0.001; T60 vs. T90, *p* = 0.031), whereas in the P2 group the VSC level remained stable at subsequent measurement at T60 and T90 (*p*-values, T30 vs. T60, *p* = 0.144; T60 vs. T90, *p* = 0.999). The differences between groups were statistically significant at T60 (P1, 1.45 ± 0.79 vs. P2, 0.93 ± 0.61; *p* = 0.007) and T90 (P1, 1.83 ± 0.61 vs. P2, 0.98 ± 0.73; *p* < 0.001; Figure 4).

### Oral health index (OHI)

**Figure 5:**
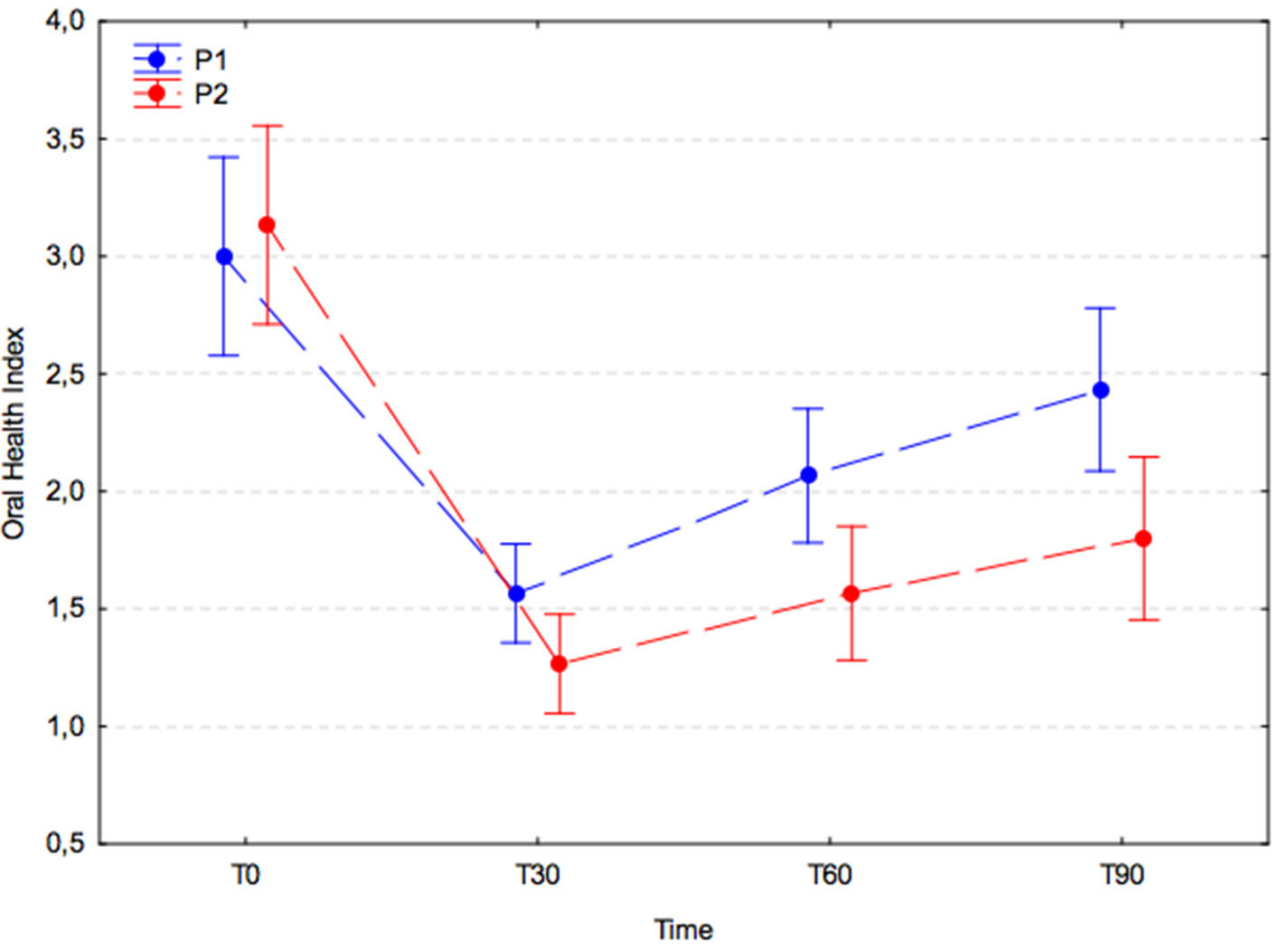
Oral health index (arithmetic mean with 95% confidence interval) in dogs receiving edible treats containing placebo (P1 group, blue) or *A. nodosum* (P2 group, red) for 90 consecutive days. Dotted lines connect data obtained at specified time points for each experimental group.

OHI values were similar in both experimental groups at T0 (P1, 3.0 ± 1.2; P2, 3.1 ± 1.1) and dropped significantly in the 30 days after the dental procedure (P1, 1.6 ± 0.5; P2, 1.3 ± 0.6; *p* < 0.001). Subsequently, the OHI increased significantly in the placebo group (P1) at T60 (2.1 ± 0.7; T30 vs. T60; *p* = 0.023) and remained stable at T90 (2.4 ± 0.9; T60 vs. T90; *p* = 0.240), whereas in the *A. nodosum*-treated group (P2) it remained unchanged at T60 (1.6 ± 0.9; T30 vs. T60; *p* = 0.505) and T90 (1.8 ± 1.0; T60 vs. T90; *p* = 0.792). The increases in OHI were > 30% larger in the placebo group than in the treated group at T60 and T90; however, the differences were not significant (p = 0.090; Figure 5).

### Gingival bleeding index (GBI)

**Figure 6:**
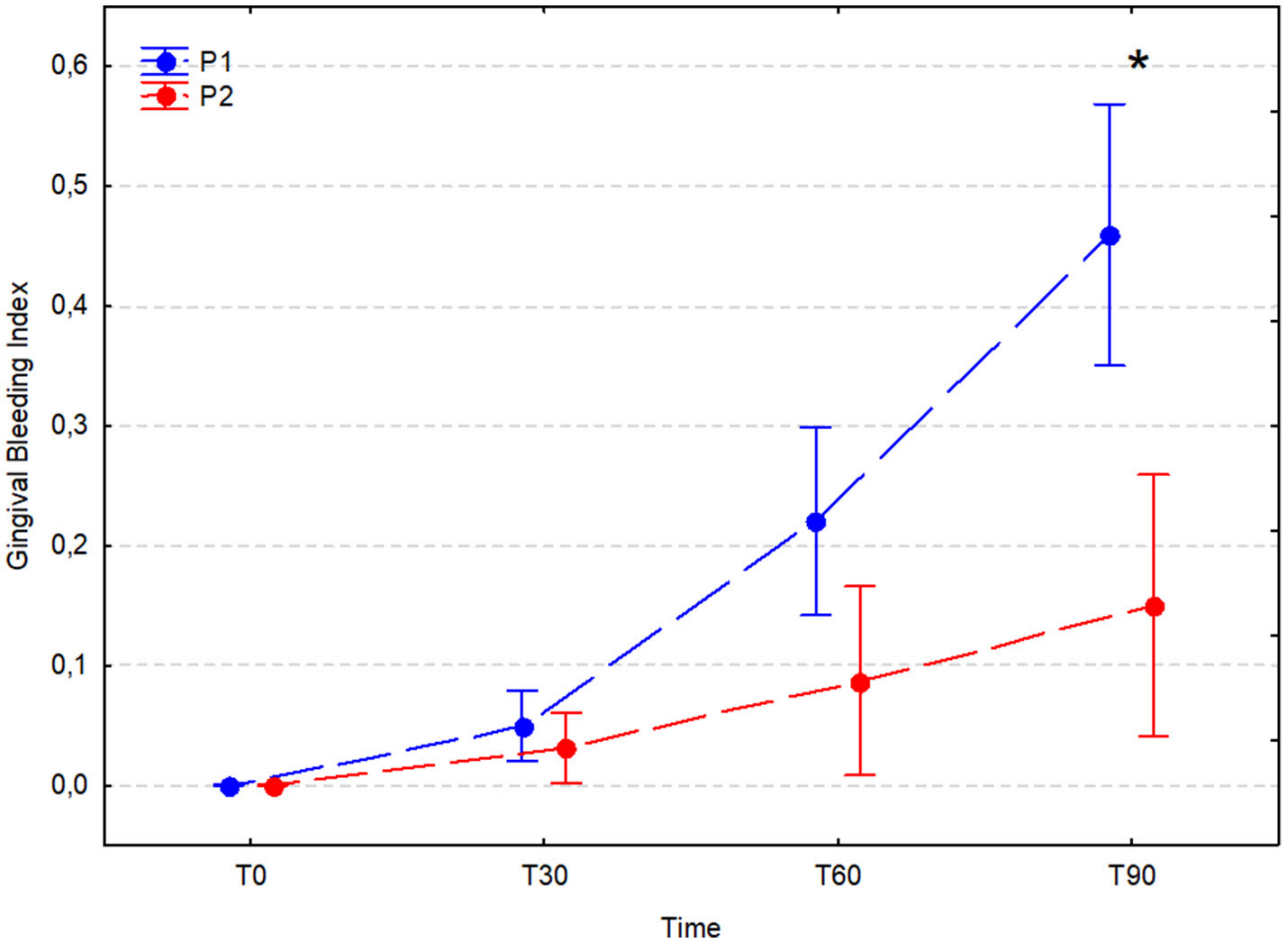
Average gingival bleeding index (arithmetic mean with 95% confidence interval) in dogs receiving edible treats containing placebo (P1 group, blue) or *A. nodosum* (P2 group, red) for 90 consecutive days. Asterisk (^*^) denotes significant intergroup difference at a given time point. Dotted lines connect data obtained at the specified time points in each experimental group.

The GBI did not increase significantly in either experimental group during the 30 days after the dental procedure; however, after 60 and 90 days it increased significantly in the placebo group (T60 P1, 0.22 ± 0.22; T30 vs. T60, *p* < 0.001) and (T90 P1, 0.46 ± 0.29; T60 vs. T90, *p* = 0.031). In the *A. nodosum*-treated group (P2), the bleeding index was stable between T0 and T30 (*p* = 0.990), T30 and T60 (*p* = 0.803), and T60 and T90 (*p* = 0.681); however, it was significantly higher at T60 and T90 than at T0 (*p* < 0.001 for comparisons with both time points). A significant difference between the P1 and P2 groups was noted at T90 (P1, 0.46 ± 0.29 vs. P2, 0.15 ± 0.30; *P* < 0.001; Figure 6).

## Discussion

To reduce the number of dogs that suffer from periodontal diseases, numerous oral hygiene modalities have emerged which can be classified into two major forms of treatment: passive and active (30). Despite numerous clinical attempts to evaluate the effectiveness of both passive and active methods of periodontal disease prevention, only a limited number of studies have been conducted according to evidence-based medicine (EBM) criteria to evaluate the effectiveness of home care for the prevention of periodontal disease in pets (31). Although numerous products claiming to be effective in maintaining oral health are commercially available, there is limited evidence of plaque and/or calculus control for the majority of these. The most desirable data are those generated by the highest grades of EBM: grade I, which are obtained from properly designed, randomized, controlled studies, conducted on target species; and grade II where evidence is obtained from properly designed, randomized, controlled studies conducted on the target species in a lab or research colony (32, 33).

One passive method of periodontal disease prevention is the oral administration of supplements containing the brown algae, *A. nodosum*, which is reported to improve OHI in dogs, cats (14), and humans (21). While the mechanism of action for this product is unknown, it has been shown to assist host immune function. One study showed that it improved dendritic cell maturation (34), and several others have demonstrated a immunostimulatory activity for *A. nodosum* like induce the secretion of tumor necrosis factor-α (TNF-α) and granulocyte colony-stimulating factor (G-CSF), nitric oxide from RAW264.7 cells or increased splenic natural killer cell activity against YAC-1 cells in mice (35–37). Accordingly, the current double-blind, randomized, placebo-controlled, single-center study aimed to deliver more reliable evidence of the oral effects of brown algae in dogs.

Miniature and toy dog breeds are predisposed to periodontal disease (38). Long-term observations of small breeds (body weight <12 kg) have revealed the establishment and progression of periodontal disease, despite the implementation of passive (dry kibble diet) and active (teeth brushing every other day) oral hygiene measures. In one evaluation of 52 Miniature Schnauzers, only one dog had teeth that did not exhibit progression to periodontitis after 60 weeks of observation (39). The present study was conducted using five different breeds; however, the majority (*n* = 48) belonged to two breeds: Miniature Schnauzer and Chihuahua. The remaining three breeds, Japanese chin, Pomeranian, and WHWT, were also small and toy breeds. The assumed predilection for rapid plaque accumulation, which is associated with the development of periodontal disease, was clearly evidenced by the rapid increase in combined PI in the placebo-treated group (30 days after the oral procedure the mean combined PI was 0.96, reaching 2.71 after 90 days). In addition, in the placebo group, which received a dry kibble diet as the only passive oral hygiene method, dental deposits accumulated significantly more quickly than in dogs treated with *A. nodosum*. Further, the oral health condition of the placebo group deteriorated within a relatively short period of time.

There were marked differences between the placebo and active groups for all investigated parameters and at all evaluated time points; however, not all differences were statistically significant. *A. nodosum* administration resulted in reduced plaque formation; this was statistically significant on day 90. Regarding calculus formation, the calculus index in the *A. nodosum*-treated group was significantly lower at all measurement points. VSC levels were 24% lower in the *A. nodosum*-treated group on day 30, and 35 and 46% lower on days 60 and 90, respectively; the differences on days 60 and 90 were statistically significant. The differences between the two groups in OHI were not statistically significant at any evaluated time point, despite an 18% lower OHI on day 30 in *A. nodosum*-treated dogs, and values 23 and 25% lower on days 60 and 90, respectively. This is in contrast with the findings of our previous study conducted on dogs and cats supplemented with *A. nodosum* seaweed for 42 days after an oral hygiene procedure, which resulted in a greater than 2-fold decrease in OHI in dogs and cats compared with control animals (20). The reason for this difference between the findings of the studies may be attributable to the larger population of animals assessed in the current study compared with the previous investigation (*n* = 60 vs. 12). Additionally, in the present study, all evaluated dogs were of small breeds, whilst the previous group had a wide range of breeds and body weights (7–40 kg). Finally, OHI is not sufficiently sensitive to detect changes in the early stage of periodontal disease; therefore, no significant difference was observed in this short study.

In the present study, VSC concentration was selected as a study criterion as VSCs are potential etiopathogenic factors in periodontal diseases (40). The results indicated a significant difference in VSC concentration when the product was used for 60 and 90 days; however, during the first 30 days after professional dental cleaning, there were no differences between study groups. VSC concentration was measured in the entire maxillary facial (buccal and labial) gingival margin. There is evidence of more intensive calculus accumulation in the area of caudal maxillary premolar teeth, due to their location adjacent to the salivary gland duct openings (27). This may explain the equal accumulation of deposits at this location in both groups during the first observation period. Nevertheless, the buccal area of the maxillary premolars has not been identified as a site with a predilection for rapid development of periodontitis (39). Significant differences in VSC concentration in the test group highlight the positive role of *A. nodosum* in prevention of some negative aspects of periodontal diseases.

The results of this study suggest that *A. nodosum*-containing supplements, used as a passive mode of oral hygiene in the form of dry kibble, have beneficial properties. *A. nodosum* has been shown to reduce plaque by up to 87% and calculus by up to 68% in humans (21); however, its exact mechanism of action remains unclear. *A. nodosum* is rich in natural compounds (41) that are postulated to interfere with bacterial growth and accumulation. These, and other compounds indigenous to the alga, as well their metabolites, are absorbed in the intestine and may be excreted into the oral cavity via the saliva. The influence of brown algae on oral health may be attributable to its antioxidative effects or its sulfur-containing bioactive carbohydrates (i.e., ascophyllans and/or polyphenols/phlorotannins), which may induce the production of short-chain fatty acids in the gut (41–45). Some studies have found that fucoidans/ascophyllans have antimicrobial effects due to their influence on the adhesion of microorganisms (46–48). The antifouling effects of various aquatic organisms, including *A. nodosum*, have been studied and a patent relating to these effects on aquatic surfaces has been filed (Patent 51) (49).

Despite its unknown mechanism of action, supplementation with *A. nodosum* seaweed was found to slow the progression of periodontal disease. Based on this study, its activity cannot be directly compared with the effectiveness of various mechanically active treats or bars, or other locally active products, as *A. nodosum* does not appear to act locally. In terms of the putative antibacterial effects of *A. nodosum*, products containing this alga may be compared with zinc salts, essential oils, or tea polyphenols (50); however, in order to confirm this, studies following evidence-based medicine standards are required. These products are suggested to prevent plaque formation and halitosis when administered directly into the oral cavity. Similar local activity observed on administration of oral treats or bars is attributable to mechanical effects caused by their particular textures. Many of these products are also fortified with substances, such as hexametaphosphate, sodium tripolysulfate, zinc ascorbate, and sodium pyrophosphate, which have calcium-chelating properties. Calcium chelation prevents the calcification of plaques and calculus formation. Mechanical and chemical mechanisms of action can reduce calculus formation by 20–80% depending on the type of substance used and the duration of administration (from 4 weeks to 4 months) (51–57). In our study, the calculus index was reduced by 35% on day 30, and this reduction was stable over the remaining treatment period. This may be attributable to the chemical action of *A. nodosum* or metabolites formed during ingestion of the product. The small size of the kibbles, and the small number consumed, is unlikely to result in mechanical effects.

All dogs completed the 90-day study in good condition and without any clinically observed health problems. We observed an increased body weight in the evaluated dogs (both groups) during the first 30 days of observation, which was probably caused by the change in their diet. According to the owner questionnaire, there were no signs of indigestion during the study period and all dogs accepted the provided food. None of the reported observations could be considered negative effects of the product.

One of the most important inclusion criteria was the level of thyroxine in the assessed population of dogs. *A. nodosum* seaweed has a balanced composition of different nutrients, including a high iodine content (450–1,000 ppm); the edible treats provided 16–32 μg iodine per kg dog per day. The safe upper daily limit of iodine for dogs is 170 mcg/kg (58) and dogs received an average of 18.7 mcg/kg/day during this study. Accordingly, to screen for negative effects on health due to the high iodine content of the active product, thyroxine levels were analyzed in blood samples from the dogs. For animals with inappropriate thyroid hormone regulation, or those on dietary or pharmaceutical therapy for hyper or hypothyroidism, caution should be exercised, as this product does contain significant amounts of iodine. It was assumed that, in dogs with normal thyroid function characterized by normal levels of thyroxine in serum, these amounts of iodine would not induce any adverse effects. This conclusion was based on a study demonstrating that no toxic effects occurred when alga was included in the diet at concentrations of up to 15% (59).

In conclusion, 90-day supplementation of dogs with kibbles containing *A. nodosum* seaweed resulted in significant improvements in several dental health indices and was beneficial for the prevention of plaque and calculus formation after a prophylactic dental procedure. The resulting reduction of levels of VSCs suggests that *A. nodosum* supplementation may be effective for long-term prevention of halitosis as well as for maintenance of good oral health.

## Author contributions

JG authored the study design, conducted the scoring for the research, and supervised the medical part of the study. MJ conducted the statistical analyses and contributed to the discussion. US proposed that the study should last for 90 days and helped to review the text. KJ practically managed the study and oversaw the blinding of investigators and randomization of the participants. EK participated in conducting experiments.

### Conflict of interest statement

The study design and budget of the entire project was financed by Sweden Care, the manufacturer of the tested product. In contract signed prior to start the studies it was agreed that the participating veterinarians have the right to publish the results in a publication written by Scientific Director JG. The SDC will contribute with comments and has the right to read and comment the final manuscript before publication.
